# Flow cytometry remission by Ig light chains ratio is a powerful marker of outcome in multiple myeloma after tandem autologous transplant: a real-life study

**DOI:** 10.1186/s13046-016-0324-0

**Published:** 2016-03-19

**Authors:** Iole Cordone, Francesco Marchesi, Serena Masi, Valentina Summa, Francesco Pisani, Roberta Merola, Giovanni Cigliana, Giulia Orlandi, Svitlana Gumenyuk, Francesca Palombi, Atelda Romano, Antonio Spadea, Daniela Renzi, Elena Papa, Marco Canfora, Laura Conti, Maria Concetta Petti, Andrea Mengarelli

**Affiliations:** Clinical Pathology, Regina Elena National Cancer Institute, Rome, Italy; Hematology and Stem Cell Transplant Unit, Regina Elena National Cancer Institute, Rome, Italy; Scientific Direction, Regina Elena National Cancer Institute, Rome, Italy; Clinical Pathology, Regina Elena National Cancer Institute, Via Elio Chianesi 53, 00144 Rome, Italy

**Keywords:** Multiple myeloma, Minimal residual disease, Flow cytometry remission, Light chains ratio, Autologous stem cell transplant

## Abstract

**Background:**

The achievement of complete response (CR) significantly correlates with a better clinical outcome in multiple myeloma (MM) patients treated with autologous stem cell transplant (ASCT). The depth of response is one of the most relevant factors to predict patient’s outcome, however the definition of CR through standard criteria has shown several limitations.

**Methods:**

In this study we evaluated the minimal residual disease (MRD) in 50 consecutive MM patients who underwent an up-front tandem ASCT in our center, using a single-tube six-colors flow cytometry assay (FC) based on intra-cytoplasmic immunoglobulin (cy-Ig) light chains ratio evaluated on patient-specific plasma cells (PC) immune profile, in a real-life setting.

**Results:**

With a sensitivity up to 10^−5^, clonal-PC were documented by FC in 36.4 % (12/33) of patients in conventional CR after second transplant. The number of flow MRD-negative patients significantly increased after induction and first ASCT, but not between first and second transplant. The 5-years progression-free survival (5ys-PFS) of flow MRD-negative patients after second transplant was significantly better than patients who remained MRD-positive considering both all patients (5ys-PFS: 70 % *vs* 5 %) and patients in CR according to standard criteria (5ys-PFS: 67 % *vs* 0 %).

**Conclusions:**

FC remission through cy-Ig light ratio on PC sub-populations is a sensitive, highly informative, low-cost and routinely applicable MRD assay, a powerful tool in treatment response evaluation and a crucial marker of outcome in MM.

## Background

Minimal residual disease (MRD) monitoring by multiparameter flow cytometry (FC) is a powerful tool for treatment efficacy and outcome prediction in hematological malignancies and several reports are focusing on its clinical relevance in multiple myeloma (MM) patients [1–9], in order to better subcategorize the quality of response compared to other approaches [10–13]. However, the identification and standardization of a sensitive, reproducible and clinically relevant analytical approach is still under consideration for international consensus [14, 15] and therefore, FC is not routinely performed in the current clinical practice, being most of MM MRD assessment by FC reported into controlled clinical trials [16–18].

In this study, we developed a 6-colors single-tube FC assay for MRD monitoring, based on intra-cytoplasmic immunoglobulin (cy-Ig) light chains ratio on patient-specific plasma cell (PC) immune profile. This approach was employed on 50 consecutive MM patients who underwent an up-front tandem autologous stem cell transplant (ASCT) and our 5 years follow-up analysis showed that this inexpensive and sensitive test can efficiently predict MM outcome even in a real-life single center setting.

## Methods

### Patients

From January 2006 to January 2012, 50 consecutive MM patients, diagnosed according to International Myeloma Working Group criteria [19] and treated in our Institution with an up-front tandem ASCT-based therapeutic program entered the study. Baseline patient characteristics are described in detail in Table 1. Two cases of IgD myeloma have also been included, previous studies suggesting that this myeloma subtype, treated with ASCT, shows a similar prognosis as other myeloma patients [20]. We have also included a plasma cell leukemia, considering that its clinical course was similar to other myeloma patients in which MRD negativity was never achieved. All patients were stratified at diagnosis on the basis of cytogenetic assessment [21]. Median follow-up was 60 months (24–108). All patients provided written informed consent for scientific purposes.

**Table 1:** Baseline patient characteristics (*n* = 50)

Parameter	N (%)
Sex, male	29 (58)
Median age at diagnosis, yrs. (range)	56 (41–68)
Diagnosis	
IgG	20 (40 %)
IgA	11 (22 %)
IgD	2 (4 %)
Light chain	16 (32 %)
Plasma cell leukemia	1 (2 %)
Durie and Salmon stage	
IA	2 (4)
IIA	17 (34)
IIIA	29 (58)
IIIB	1 (2)
Na (^a^)	1 (2)
ISS stage	
1	26 (52)
2	17 (34)
3	5 (10)
Na (^a^)	1 (2)
Cytogenetic analysis [21]	
High risk	6 (12 %)
Intermediate risk	8 (16 %)
Standard risk	35 (70 %)
Na (^a^)	1 (2 %)
Induction treatment	
VAD (^b^)	23 (46)
Novel agents (^c^)	27 (54)
Up-front tandem ASCT (^d^)	50 (100)

(^a^) *Na* not available. (^b^) *VAD* Vincristine, Adryamicin, Dexamethasone (2 courses). (^c^) Novel agents: Bortezomib-based regimes (*n* = 21, 3 courses), Immunomodulators only (*n* = 6, 3 courses). (^d^) *ASCT* autologous stem cell transplant

### Flow cytometry

At diagnosis, FC analysis of the PC surface markers was performed on erythrocytes-lysed EDTA-anti-coagulated bone marrow (BM) samples using a 6-colors panel of antibodies (Fitc/PE/PerCP/PE-Cy7/APC/APC-Cy7) and the “Duo-lyse” program of the Becton Dickinson Bioscience (BDB) Lyse-Wash-Assistant according to the 1) CD28/CD138/CD45/CD38/CD33/CD20; 2) CD38/CD138/CD45/CD56/CD117/CD19 antibodies combinations. The PC Surface-Aberrant-Markers (SAM) were used as patient-specific immune profile to document cy-Ig light chains restriction utilizing a single-tube 6-color intra-cytoplasmic staining: 3) cy-Ig lambda/cy-Ig kappa/CD19/CD38/SAM+/CD45 at diagnosis and, for MRD monitoring, after induction and at day +100 after both first and second transplant. For cytoplasmic staining cells were washed twice in PBS prior to staining, fixed and permeabilized using the Cytofix & Cytoperm kit (BDB) according to manufacturer’s recommendations, incubated with the monoclonal antibodies cocktail for 20 min at 4 °C, washed in PBS and promptly acquired. All the antibodies were from BDB but CD28, CD33 and CD138 from Beckman Coulter. Light scatter and CD38 signal was used for PC gating. A minimum of 2x10^3^ PC were acquired. If not available, the whole stained sample was consumed. A sample was considered suitable form MRD evaluation when at least 150 PC were counted. Markers expression was reported as percentage of positive cells within the CD38-positive population. To differentiate between normal and neoplastic PC, the kappa/lambda ratio was evaluated on the whole CD38-positive PC population and on any of the CD38 sub-populations. Patients were considered FC positive for residual disease (flow MRD-positive) when a PC kappa/lambda ratio either <0.5 or >4.0 was documented [22]. The CD19-positive BM lymphocytes (identified as CD45-strong expression and intermediate side-scatter signals), were utilized as internal control for kappa/lambda ratio staining.

Overall, a total of 200 BM samples were processed within 24 h from collection for MRD evaluation using a BDB FACSCanto flow cytometer with FACSDiva software.

### Statistics

Data were analyzed using Statistical Package of Social Sciences software (SPSS, version 17.0, Chicago, USA). The correlation between treatment response by standard criteria [23] and FC assessment according to check-point of the therapeutic program was performed using the Chi-square test (Fisher or Pearson) and Anova test for categorical and quantitative variables, respectively. Progression-free survival (PFS) curves were calculated by the Kaplan-Meier method and compared using the two-side log-rank (Mantel-Cox) test. Two-sided *P* values <0.05 were considered as statistically significant.

## Results

The PC surface aberrant markers expression documented at diagnosis is shown in Table 2. For MRD evaluation, gating on CD38-bright population in combination with the side scatter, a median of 2317 (range 187–59609) PC was acquired and analyzed on up to 3.0 x 10^6^ total BM cells (median total events acquired: 1.01 x 10^6^, range 1.19 x 10^5^–3.02 x 10^6^), with a median of 0.2 % (range 0.03–53) PC out of total BM leucocytes, at a sensitivity level up to 10^−5^. Eleven samples (5.5 %) were inadequate (hemodiluted) for MRD analysis and not considered for further analysis.

**Table 2:** PC aberrant markers expression at diagnosis and during MRD monitoring by flow cytometry assessment

	Diagnosis	Follow-up		Follow-up	
PC surface aberrant markers (SAM)	SAM expression (% of patients)	MRD-negative by cy-Ig light chains ratio on SAM percentage above the normal threshold ^6, 7^ (Putative surface MRD-positive) (% of samples)		MRD-positive by cy-Ig light chains ratio on SAM percentage below the normal threshold ^6, 7^ (Putative surface MRD-negative) (% of samples)	
CD19neg	98 %	CD19neg ≥30 %	2.5 %	CD19neg <30 %	26 %
CD45weak/neg	63 %	CD45weak/neg ≥6 %	38 %	CD45weak/neg <6 %	39 %
CD56pos	74 %	CD56pos ≥15 %	15 %	CD56pos <15 %	27 %
CD117pos	41 %	CD117pos ≥1 %	0 %	CD117pos <1 %	*na*
CD33pos	26 %	CD33pos ≥6 %	60 %	CD33pos <6 %	0 %

*PC* plasma cells, *SAM* surface aberrant marker, SAM% calculated within the CD38pos PC population, *MRD* minimal residual disease, *cy-Ig* cytoplasmic immunoglobulin, *na* not applicable

**Fig. 1:**
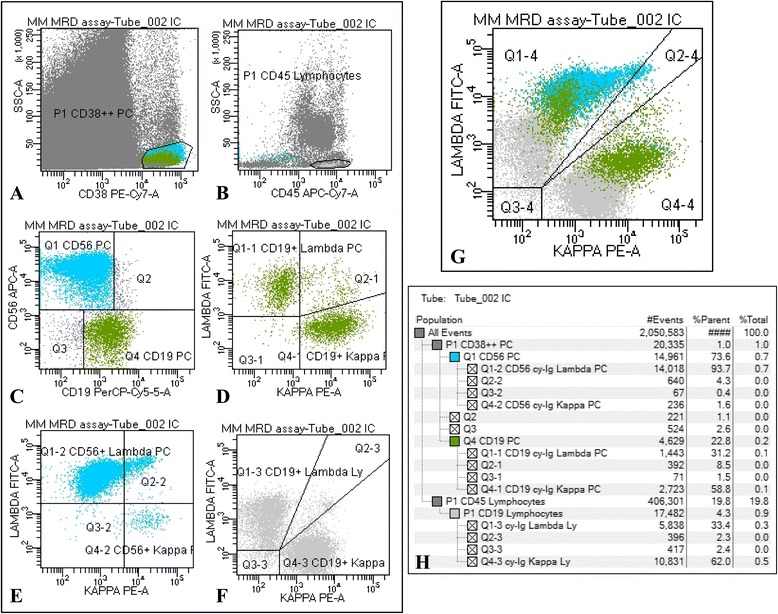
Flow cytometry gating strategy for MRD assessment: representative analysis of a bone marrow sample. **a** CD38^bright^ PC population, **b** CD45+ Lymphocytes (Ly), **c** surface membrane expression of CD19 and CD56 assessed on CD38^bright^ PC, **d** cy-Ig Kappa/Lambda *ratio* on CD19+ PC (normal; *ratio* 1.9); **e** cy-Ig Kappa/Lambda *ratio* on CD56+ PC (clonal; *ratio* 0.01); **f** cy-Ig Kappa/Lambda *ratio* on CD19+ Ly utilized as internal control (normal; *ratio* 1.8); **g** cy-Ig Kappa/Lambda *ratio* on the PC and Ly populations; **h** populations hierarchy

On the whole CD38-bright PC population, cy-Ig light chains restriction was documented in 35 % of samples after treatment. However, gating on CD38/SAM sub-populations (CD19neg, CD45weak/neg, CD56pos, CD117pos, CD33pos) alone or in combination, clonal-PC were overall identified in 62.5 % of samples after treatment, being CD117 the most informative single SAM for clonal-PC detection. Moreover, in a considerable proportion of samples with percentage of CD19neg CD45weak/neg and CD56pos PC below the normal threshold, clonal PC were easily identified by cy-Ig light chains ratio after treatment (Table 2). Co-presence of normal and neoplastic PC was observed in 100 % of MRD-positive cases (Fig. 1). Focusing to patients in conventional CR, cy-Ig light chains ratio on SAM sub-populations documented clonal-PC in 36.4 % after second transplant (12/33) and in 43.2 % of overall patients irrespective to the check-point of the therapeutic program (32/74), including BM samples with a SAM percentage below the normal threshold.

**Fig. 2:**
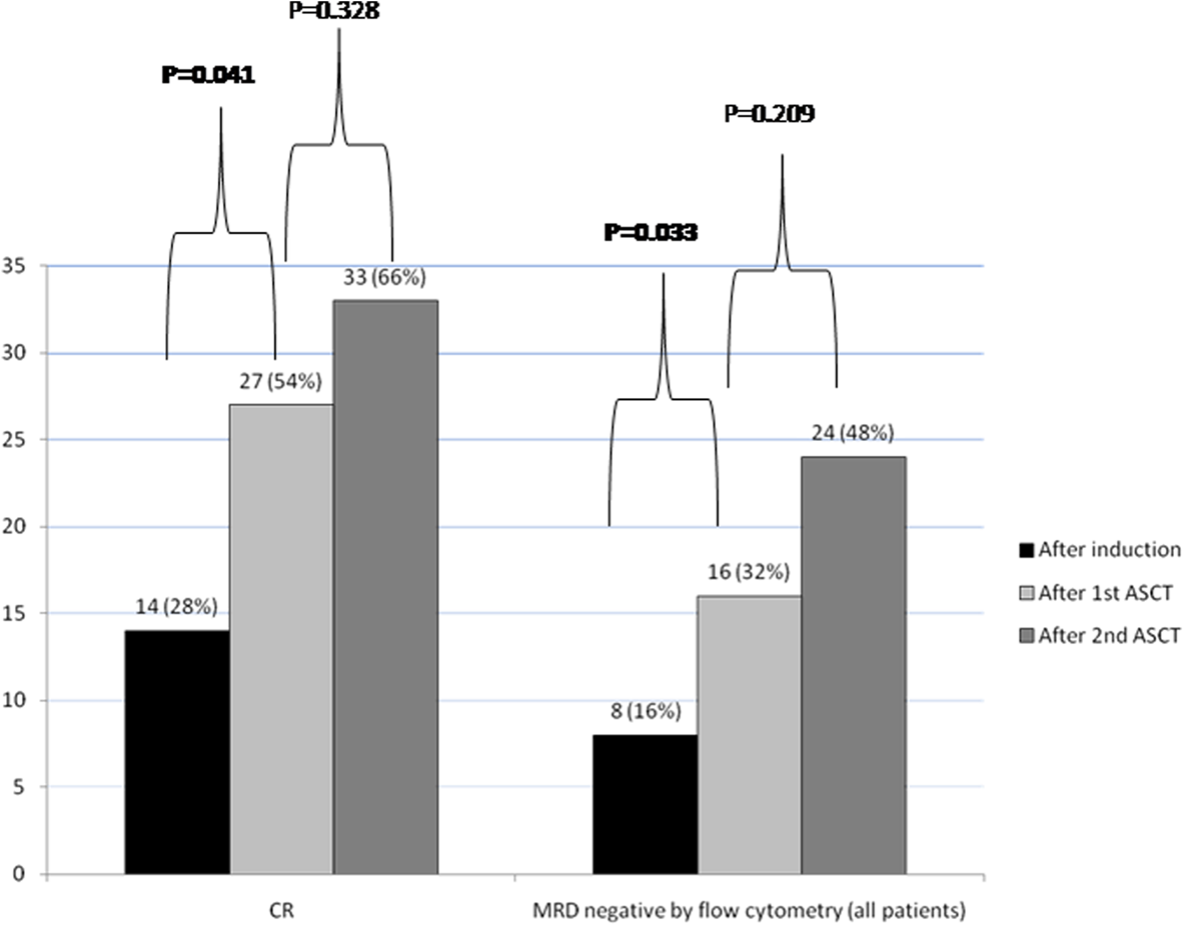
Patients in CR according to standard criteria and MRD negative from FC assessment in all check-points of the therapeutic program

**Fig. 3:**
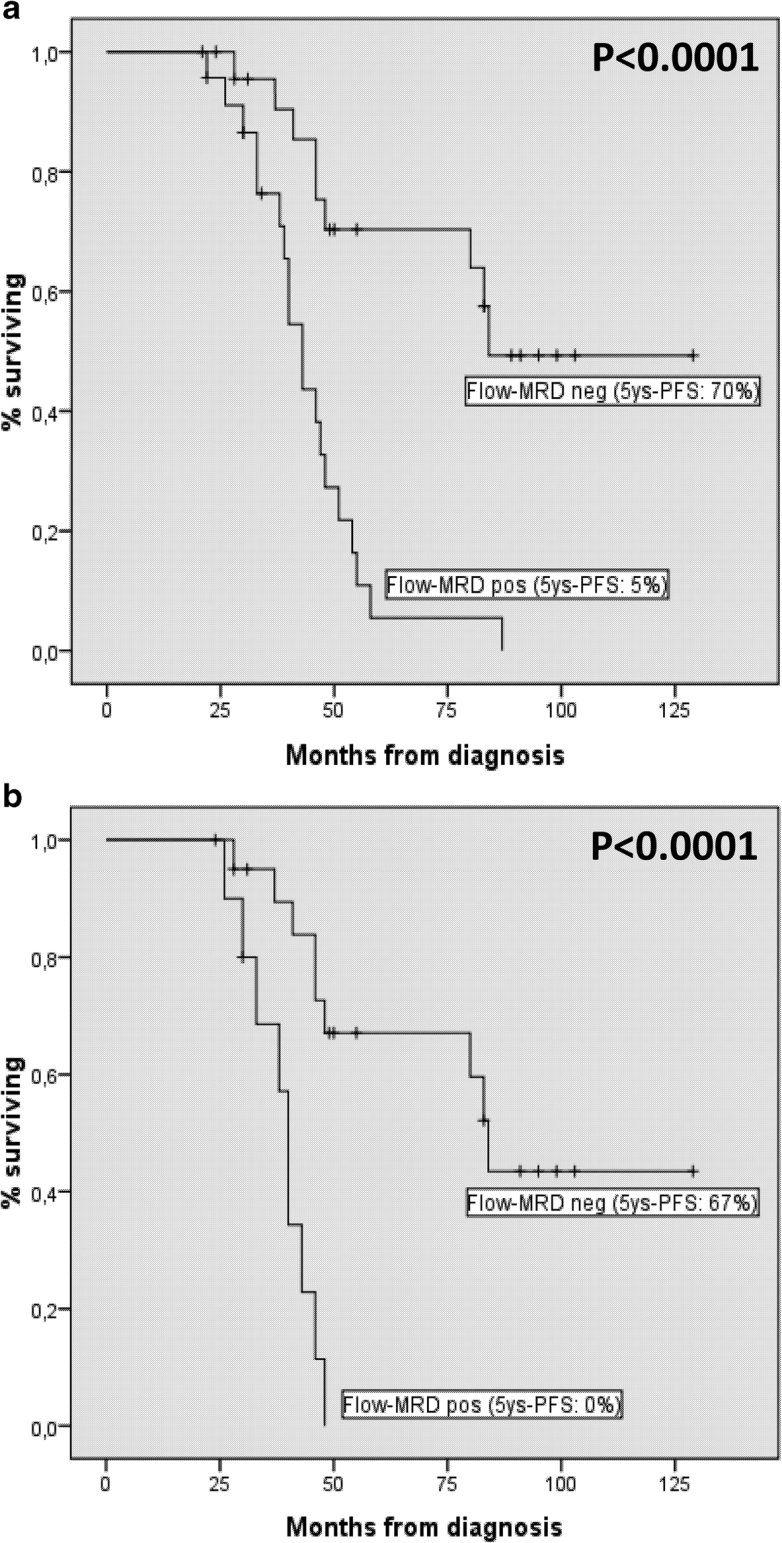
5-years PFS curves according to MRD assessment by FC after second transplant: all patients (**a**); patients in CR according to standard criteria (**b**)

A significant decrease of clonal-PC was observed between induction and first transplant (63 % *vs* 5 %; *P* < 0.0001), but not between first and second ASCT (5 % *vs* 1 %; *P* = 0.412). Treatment response by standard and FC criteria, according to check point of the therapeutic program, is shown in Fig. 2. Compared to conventional CR, a lower rate of immunophenotypic CR (flow MRD-negative) was documented in all check-points of the therapeutic program (28 % *vs* 16 % after induction, 54 % *vs* 32 % after fist and 66 % *vs* 48 % after second ASCT). The 5ys-PFS of flow MRD-negative patients after second transplant was significantly better compared to MRD-positive (5ys-PFS 70 % *vs* 5 %, respectively; *P* < 0.0001, Fig. 3a). According to flow MRD assessment, patients in conventional CR showed a significant difference in 5ys-PFS after second transplant (5ys-PFS 67 % *vs* 0 % for MRD-positive and MRD-negative patients, respectively; *P* < 0.0001, Fig. 3b). After induction, we found a significant higher rate of conventional CR and flow MRD-negative cases in patients who received a novel agents-based treatment compared with those treated with VAD regimen (10/27, 37 % *vs* 4/23, 17.4 %; *P* = 0.042 and 6/27, 22.2 % *vs* 2/23, 8.7 %; *P* = 0.039, respectively). Interestingly, this difference was also evident, but not statistically significant, after first and second transplant. Moreover, 5-ys-PFS curves of MRD-negative patients were absolutely similar among patients who received novel agents *vs* VAD as induction treatment prior first ASCT (data not shown).

## Discussion

Some of the PC SAM can be detected at low levels in healthy individuals and non-neoplastic PC can exhibit immunophenotypic characteristics overlapping with myeloma cells [6]. Loss of CD19 and CD56 expression are, in fact, commonly observed in clonal-PC population, however both features occur in subsets of polyclonal populations as well [7]. To overcome this potential pitfall, we develop an original 6-colors single-tube FC assay, based on cy-Ig light chains ratio on patient-specific PC immune profile for MRD monitoring in MM. The current manuscript describes a real-life single center study that evaluates the clinical relevance of this approach on 50 consecutive MM patients who underwent an up-front tandem ASCT, on a 5 years follow-up analysis. At the best of our knowledge, this is the first report describing an extensively, single center, flow-MRD monitoring based on cy-Ig light chains ratio evaluated on PC sub-populations in myeloma patients after transplant in a real-life setting.

Sensitivity is one of the most important factors in MRD identification and this assay has shown to discriminate between reactive and neoplastic PC in samples with very low cell count as pleural effusion and cerebrospinal MM infiltration [24]. Our selected antibody panel included all the antigens more recently recommended by The European Myeloma Network and International Clinical Cytometry Societies for PC characterization [25]. However, to discriminate between normal and malignant PC, our strategy focused on the intra-cytoplasmic light chains expression evaluated on PC sub-populations identified by the expression of normal and SAM. We found the use of light chains restriction as an important adjunct to the use of surface markers, with the capability to identify clonal-PC hidden in the context of a normal PC population in 43.2 % of patients in conventional CR, at a sensitivity up to 10^−5^. The baseline flow testing allowed to utilize a 6-color single-tube assay for MRD detection, however it is not an absolute requirement. If not performed at diagnosis, a baseline characterization can be performed after treatment to identify patient’s SAM to utilize for disease monitoring if the aberrant PC population exceeds the 10^−5^ threshold.

The sensitivity of FC for MRD detection is dependent on the number of cell analyzed and several studies pointed on the total number of cells acquired [26]. Using the light scatter and CD38 signal for PC gating, 2317 PC on a median of 1.01 x 10^6^ total events were recorded and analyzed, being 94.5 % the percentage of specimens evaluable for analysis. Acquisition of an higher number of events, up to 3–5 x 10^6^, is currently considered the best practice for MRD monitoring [26]. In MRD-negative cases, we are now increasing the number of total processed events, staining the whole BM sample and recording the whole PC population of the sample, to reach the 10^−5^ limit of detection in all samples. Check the quality of the specimen is also of primary relevance in these samples.

PC clonality was documented on a minority (35 %) of samples when the cy-Ig light chains ratio was evaluate on the whole CD38 positive population. By contrast, gaiting on minor PC subsets expressing one or more SAM, neoplastic PC were documented in the majority of sample after treatment, including BM samples with a SAM percentage below the normal threshold. Thereafter, this approach allowed determining, in a more reliable way, whether a small PC fraction with a putative aberrant phenotype (e.g., CD19neg or CD56pos) was representative of an abnormal clone or of a non-clonal PC sub-population despite the putative SAM expression, documenting that the light chains restriction is an important adjunct to the use of surface markers.

Co-presence of normal (polyclonal) and neoplastic (clonal) PC was documented in all MRD positive samples showing that, despite the tumor persistence, a fraction of normal PC emerge from the BM environment. For this reason, cy-Ig light ratio evaluated in the whole CD38 positive PC population identified clonal PC in a minority of cases after treatment, by contrast it was very informative if evaluated on the SAM sub-populations, even in samples with SAM percentage below the normal threshold. Moreover, BM clearance, evaluated through the normal *vs.* neoplastic PC ratio monitoring, could represent an early surrogate marker of treatment efficacy.

FC confirmed to be more sensitive than standard criteria. In fact, among patients in conventional CR, flow-MRD positivity was documented in 36.4 % of patients after second transplant and in 43.2 % of overall patients irrespective to the check-point of the therapeutic program, strongly supporting the relevance of cy-Ig light ratio assessment by flow MRD monitoring in this era of “next-generation-flow” [1, 7]. A strong correlation between the depth of response evaluated by FC and patient’s clinical outcome emerged from our study and, despite the relatively low number of patients included into the study, the significant difference in terms of PFS between MRD-positive and MRD-negative patients validates our approach of flow-MRD monitoring in MM.

The second ASCT has not added statistically significant advantages in term of depth of response when assessed by FC, confirming the less widely use of tandem ASCT in MM [27]. However, in clinical practice, the opportunity to perform a second ASCT is a major point of concern in MRD-positive patients after the first transplant. Further studies are warranties to address this relevant issue.

Flow MRD-negative patients after the second transplant showed a significantly better clinical outcome in terms of PFS when compared with flow MRD-positive, confirming the relevance of MRD assessment by marrow examination in all patients, irrespective to conventional CR and in agreement with previously published studies [16–18]. More relevant, the depth of response evaluated by FC was able to stratify patients in conventional CR into two groups (flow MRD-pos *vs.* neg) with a significantly different clinical outcome. Finally, as recently reported [28], also in our study the impact of post-transplant flow MRD assessment was independent of induction regimen prior transplant (i.e., novel agents *vs* VAD).

## Conclusion

MRD testing needs to be accurately defined for uniform response criteria to be utilized as an endpoint for drug approval in MM. We found the use of light chains evaluation on PC sub-populations as an important adjunct to the use of surface markers for distinguish abnormal from normal PC and a significant predictor of clinical outcome in MM patients treated with and up-front tandem ASCT. Our experience supports the role of the light chains as key markers in multicolor flow-MRD monitoring for treatment response evaluation, to maximize the therapy benefit and improve clinical management in MM in the next future.

## Abbreviations

ASCT: autologous stem cell transplant
BM: bone marrow
CR: complete remission.
cy-Ig: intra-cytoplasmatic immunoglobulin
FC: flow cytometry
MM: multiple myeloma
MRD: minimal residual disease
BDB: Becton Dickinson Bioscience
PC: plasma cells
PFS: progression-free survival
SAM: surface aberrant markers
